# A bi-articular model for scapular-humeral rhythm reconstruction through data from wearable sensors

**DOI:** 10.1186/s12984-016-0149-2

**Published:** 2016-04-23

**Authors:** Federico Lorussi, Nicola Carbonaro, Danilo De Rossi, Alessandro Tognetti

**Affiliations:** Research Center E.Piaggio, University of Pisa, Pisa, Italy; Information Engineering Department, University of Pisa, Pisa, Italy

**Keywords:** Reaching activity, Scapular girdle movement, Hand posture estimation, Scapular-humeral rhythm, Wearable sensing

## Abstract

**Background:**

Patient-specific performance assessment of arm movements in daily life activities is fundamental for neurological rehabilitation therapy. In most applications, the shoulder movement is simplified through a socket-ball joint, neglecting the movement of the scapular-thoracic complex. This may lead to significant errors. We propose an innovative bi-articular model of the human shoulder for estimating the position of the hand in relation to the sternum. The model takes into account both the scapular-toracic and gleno-humeral movements and their ratio governed by the scapular-humeral rhythm, fusing the information of inertial and textile-based strain sensors.

**Method:**

To feed the reconstruction algorithm based on the bi-articular model, an ad-hoc sensing shirt was developed. The shirt was equipped with two inertial measurement units (IMUs) and an integrated textile strain sensor. We built the bi-articular model starting from the data obtained in two planar movements (arm abduction and flexion in the sagittal plane) and analysing the error between the reference data - measured through an optical reference system - and the socket-ball approximation of the shoulder. The 3D model was developed by extending the behaviour of the kinematic chain revealed in the planar trajectories through a parameter identification that takes into account the body structure of the subject.

**Result:**

The bi-articular model was evaluated in five subjects in comparison with the optical reference system. The errors were computed in terms of distance between the reference position of the trochlea (end-effector) and the correspondent model estimation. The introduced method remarkably improved the estimation of the position of the trochlea (and consequently the estimation of the hand position during reaching activities) reducing position errors from 11.5 cm to 1.8 cm.

**Conclusion:**

Thanks to the developed bi-articular model, we demonstrated a reliable estimation of the upper arm kinematics with a minimal sensing system suitable for daily life monitoring of recovery.

## Background

Daily-life evaluation of patient’s functional performance is essential for optimal guidance in neurological rehabilitation therapy and requires unobtrusive and ambulatory monitoring systems. An accurate estimation of the hand position with respect to the sternum is important to assess the recovery induced by the treatment and to prevent compensatory movements (e.g. spine flexion or scapular elevation in reaching tasks to compensate the functional deficiency). From a technological perspective, the challenge is to obtain a reliable and quantitative evaluation with a minimal set of unobtrusive sensors.

As widely described in the kinesiology literature [1, 2], the human shoulder can be considered as a bi-articular six degrees of freedom kinematic chain. Flexion on the sagittal plane and abduction of the arm can be described as the composition of the movements of the scapular-thoracic joint or *scapular girdle* (i.e. the scapula sliding and spinning on the rib cage), the gleno-humeral joint (i.e. the humerus spinning, rolling and sliding into the glenoid cavity), and the spine. In addition, the two joints move together in a precise variable ratio, according to the *scapular-humeral rhythm* described by Crosbie et al. in [3]. In the last few decades several studies on shoulder mechanics have been performed and several models have been developed with different levels of complexity. In 1987, Högfors et al. [4] provided a significant description of the kinematics of the shoulder, based on coordinate frames fixed with bones of the scapular girdle, starting from human dissection. Two years later, A. E. Engin et al. [5] presented their “Three-Dimensional Kinematic Modelling of the Human Shoulder Complex” based on sinus cone joints and trajectories. In 1994, Van der Helm [6] proposed his finite elements model for the shoulder mechanism, introducing the concept of rolling on differentiable manifolds for joint surfaces. The following year, Happee et al. [7] presented a study on shoulder modelling including 95 muscles modelled as a third-order non linear system.

Despite the complex mechanical structure of shoulder, most research works performed the kinematic reconstruction of the upper limb by considering the shoulder as a socket-ball joint and neglecting the scapular contribution. In 2001, Prokopenko et al. [8] assessed a 7-DOF model of the upper limb where the shoulder is modelled as a socket-ball joint by using a laboratory set-up based on an electromagnetic motion tracker. Similar approaches, set-ups and results were obtained by Biryukova [9], Hingtgen [10] and Rab [11]. Few works considered the multi-articular approach. Among the others, we report the work of Retting [12] that assessed, through an optical system and skin applied markers, an advanced model of the shoulder on a large number of subjects. To the best of our knowledge, very few works combine the bi-articular approach with an ambulatory measurement set-up. A relevant example is the work of Cutti et al. [13] that estimated the scapulo-thoracic and humero-thoracic movements by using three inertial measurements units (placed on the arm, the sternum and the scapula).

To promote a step forward in unobtrusive human motion analysis, De Rossi and Veltink proposed an hybrid approach based on the fusion of data derived from inertial and e-textile sensors [14]. This strategy have since been implemented in studies and research projects to obtain patient’s performance assessments in daily life through textile integrated sensing [15, 16].

Inertial measurement units (IMUs) combine the information of accelerometers, gyroscopes and magnetometers, and are now widely used in wearable motion tracking [17–19]. The use of different IMUs, placed on connected body segments, and the additional information on the kinematic constraints enable most joint angles to be measured [20, 21].

In the last decade, several works have reported about textile-based or *e-textile* sensors for unobtrusive human motion detection. Notable contributes include [22–27]. Textile-based sensors have several advantages: low cost, lightweight, low thickness, flexibility, and the possibility of adapting them to different body structures. The main drawbacks are the reduced accuracy, the non-negligible transient time and the hysteresis. In our recent works [28–30] we employed and characterized textile-based sensors based on knitted piezoresistive fabrics (KPF) that shown reliable performances as strain and angular transducers.

In this work, we developed a new method to estimate upper arm kinematics, based on a bi-articular model of the shoulder, combining the widely used socket-ball model - which mimics the gleno-humeral joint - with an additional joint capable of describing the movement of the scapular-thoracic complex and taking into account the constraint given by the scapular-humeral rhythm. According to the model requirements, we developed a dedicated sensing shirt provided with two IMUs - to measure the arm-to-sternum orientation - and a KPF strain sensor able to to detect the scapular sliding. Note that the arm-to-sternum orientation (IMUs output) is a combination of the scapular-thoracic and gleno-humeral movements (i.e. governed by the scapular-humeral rhythm), while the strain sensor output depends only on the scapular-thoracic movement. The shoulder model we developed in this paper is a general mathematical structure based on differential roto-translational geometry whose parameters are identified in subjects using non-linear constraint optimizations. We built the bi-articular model starting from the data obtained in two planar movements (arm abduction and flexion in the sagittal plane) and analysing the error between the reference data - measured through an optical system - and the socket-ball approximation of the shoulder. The introduced method remarkably improves the estimation of the position of the trochlea (and consequently the estimation of the hand position during reaching activities) reducing position errors from 11.5 cm to 1.8 cm. The novelty of our method consisted in the embodiment of the scapular-humeral rhythm - expressed as a subject dependent relation between gleno-humeral and scapulo-thoracic movements - in the bi-articular model. This additional constraint allowed us to perform the reconstruction with a reduced sensor set (i.e. two IMUs and a mono-dimensional textile-integrated sensor) thus increasing the potential usability in daily-life.

## Methods

### The scapular-humeral rhythm

The scapula and humerus move simultaneously in a precise variable ratio: the *scapular-humeral rhythm* (Crosbie et al., [3]). The proportion of such movements varies according to the instantaneous position of the kinematic chain. The scapular-humeral rhythm is highly subject dependant through the particular morphology of the joint surfaces and the mechanical properties of the periarticular tissue. External loads may also influence the scapular-humeral rhythm.

A widely accepted approximation, described in [1], summarises the arm abduction and flexion on a sagittal plane as follows:

- In the initial phase of the arm abduction, from 0° to 100°−110°, the movement of the scapula is overall about 10°−20°, while the humerus joint moves 90° with respect to the scapula. At the threshold of 100°−110°, the gleno-humeral joint goes into the closed pack position. The remaining abduction of the arm, up to 150°, is entirely due to a scapular movement. In the final step, the arm reaches the vertical position through a hyper-extension of the spine.
- The arm flexion is composed of a spinning movement of the gleno-humeral joint of about 60° along with a 60° sliding of the scapula on the rib cage through the compliance of the subscapularis muscle. Again, the missing 60° necessary to reach the vertical position are due to spine movements.

The ratio between scapular and gleno-humeral movements can be expressed in a mathematical form as:

(1) $$ \left \{ \begin{array}{l} \psi_{a}=\frac{2}{11} \psi_{arm} \qquad 0^{\circ} \leq \psi_{arm} \leq 110^{\circ} \\ \psi_{a}= \psi_{arm}-90^{\circ} \qquad 110^{\circ} \leq \psi_{arm} \leq 150^{\circ} \end{array}\right.   $$

and

(2) $$ \phi_{a}= \frac{1}{2} \phi_{arm},   $$

where *ϕ*_*arm*_,*ψ*_*arm*_ represent the flexion on a sagittal plane and abduction angles of the upper arm and *ϕ*_*a*_,*ψ*_*a*_ are the abduction-adduction and the intra-extrarotation angles of the scapula (i.e. under the hypothesis that the coordinate systems for sternum, scapula and humerus return *ϕ*_*arm*_=0 and *ψ*_*arm*_=0 in anatomical position).

If the relations 1 and 2 were exact and not dependent on the particular subject, the measurement of *ϕ*_*arm*_,*ψ*_*arm*_ would be sufficient to describe the whole shoulder movement. Since the body structure of a particular subject influences the mechanics of his/her shoulder, relationships 1 and 2 are approximations which may lead to significant errors. However, we used the described relations as the starting point for the personalised refinement of the shoulder mechanics of individual subjects.

### Instrumentation

**Fig. 1:**
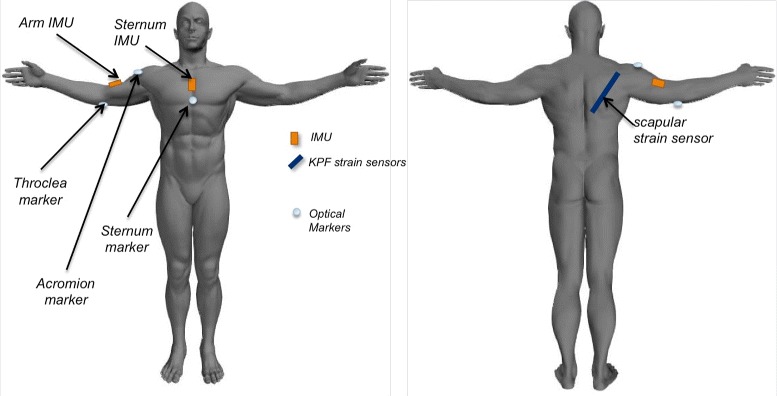
Sensor location (strain sensor and IMUs), placed on the sensing garment and used to evaluate the shoulder movement. The system is completed with the markers of the optical acquisition system (on the trochlea, the acromion and the sternum)

We collected the data derived from two IMUs (MTw produced by XSens [31]), placed on the sternum and arm respectively, and a textile-based strain sensor positioned on the back, from the spine to scapula, as shown in Fig. 1. The wearable sensors were integrated in a sensing shirt specifically produced and tested for clinical applications in stroke recovery, as we have previously described in [15, 16]. Simultaneously, we measured the 3D position of repere points (sternum, acromion, medial epicondyle of the trochlea of humerus) through an optical system (Smart DX 100 produced by BTS Bioengineering [32]) according to the marker set-up shown in Fig. 1.

The MTw used in our experiments are miniature IMUs containing triaxial accelerometers, gyroscopes and magnetometers. The MTw applies proprietary fusion algorithms to perform real time estimation of the sensor orientation. The orientation and the raw sensor data (accelerometer, gyroscope, magnetometer) are sent to a remote receiver through a wireless link. We performed the IMU to segment alignment by asking the subject to stand upright in the anatomical position with the arms neutral beside the body. The transformation from the sensor to the body segment was then calculated by matching the sensor orientations in the global frame with the know orientations of the upper arm and sternum in the considered anatomical pose, as indicated by the sensor producer in [33]. We evaluated the arm flexion on a horizontal plane (*θ*_*arm*_) and abduction (*ψ*_*arm*_) from the relative orientation of the arm IMU with respect to the sternum one.

The strain sensor was made of a knitted piezoresistive fabric (KPF) produced by Smartex [34–36]. As we demonstrated in [27], the electrical resistance of a single layer KPF sample (*R*) can be approximated, for small elongations, as a linear function of the sensor length (*l*) and curvature (*Δα*):

(3) $$ R \approx k_{1}\, l - k_{2} \, \Delta \alpha  $$

where *k*_1_,*k*_2_ are positive constants that depend on the properties of the sensing textile. In this work, we used the KPF strain sensor to detect the scapula sliding and, given the sensor configuration shown in Fig. 1, we considered negligible the contribute of the curvature (*Δα*) in Eq. 3.

### Data collection and initial analysis

Five volunteers without any pathology in their shoulders were asked to repeat (five times) two different movements involving the scapular girdle:

- flexions of the arm on a sagittal plane in the range 0°−120° (starting from the anatomical position),
- abductions of the arm on a frontal plane in the range 0°−150° (starting from the anatomical position).

We fed the socket-ball model of the shoulder with the IMU data (i.e. the arm horizontal flexion and abduction angles) to estimate the trochlea position thanks to the knowledge of the length of the anatomical segments. We then compared the estimated trochlea trajectories with the reference trajectories obtained by the optical system through the trochlea marker.

In the following sub-sections we will analyse the errors introduced by the socket-ball approximation in the described arm movements. This analysis will represent the starting point for the development of our refined bi-articular model of the shoulder.

#### Flexions on a sagittal plane

**Fig. 2:**
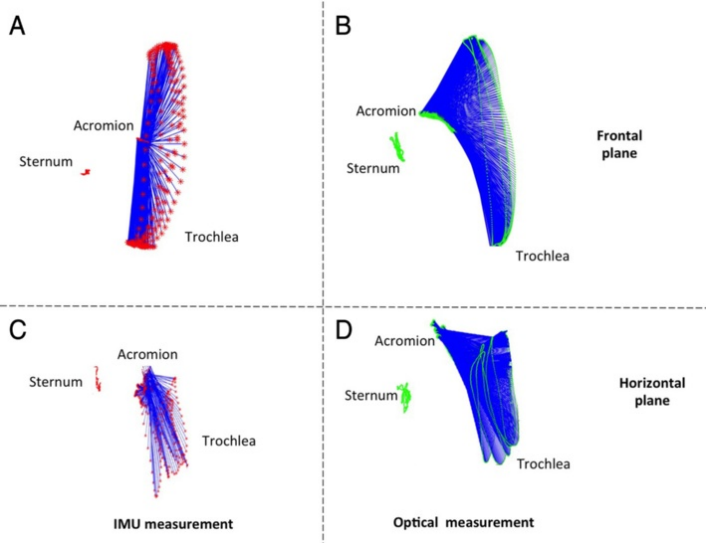
Shoulder flexion movements reconstructed by the IMU data feeding the socket-ball model (*left part*) and by the optical system (*right part*). In the *upper part*, the scapular girdle kinematics is projected on a frontal plane, in the *lower part* the projection is on a horizontal plane

**Fig. 3:**
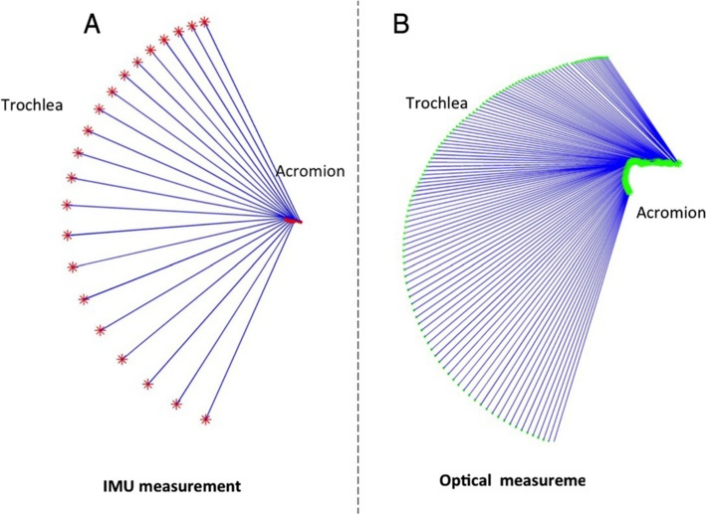
Flexion movement after removal of the sternum oscillation. Socket-ball model fed by IMU data (*left*) and optical reconstruction (*right*)

The trochlea trajectory obtained by the socket-ball model was plotted in projection on a frontal plane (Fig. 2a). The socket-ball model returned circular trajectories with the center in a point that undergoes small oscillations. These oscillations can be explained as a reaction of the trunk to the arm movements induced to maintain the balance of the center of mass and are more evident in Fig. 2c where the estimated trochlea trajectory is projected on a horizontal plane. After having mathematically removed the trunk oscillations, by subtracting the sternum movement, we obtained a perfect circular trajectory for the trochlea (Fig. 3a). Figure 2b and d shows the acromion and the trochlea reference trajectories detected by the optical system. Figure 3b, again obtained after removing the sternal oscillation, highlights the difference with respect to the socket-ball case (i.e. the reference trajectory of the trochlea is not circular). The discrepancy is above all due to the scapular movements that are neglected in the socket-ball model. We then interpolated the acromion trajectory with an elliptic arc, by committing errors less than 1 cm. Subsequently, we interpolated the trochlea trajectory as a generalized cycloid with a constant radius (i.e. the length of the humerus) and the center moving on the ellipse spanned by the humeral head (note that the humeral head is in a fixed position with respect to the acromion). The differences between the socket-ball model reconstruction and the reference data, computed for fixed angles on the repetitions of the flexion movements, are reported in Table 1.

**Table 1:** Average errors and standard deviations of the socket-ball estimation as a function of the flexion angle (tests on five subjects, five times)

Flexion angle	Error (cm)
0°	0.2±0.2
20°	1.2±0.6
40°	4.2±1.6
60°	6.4±0.9
80°	7.2±0.6
100°	8.2±0.7
120°	10.0±0.9

#### Abductions on a frontal plane

**Fig. 4:**
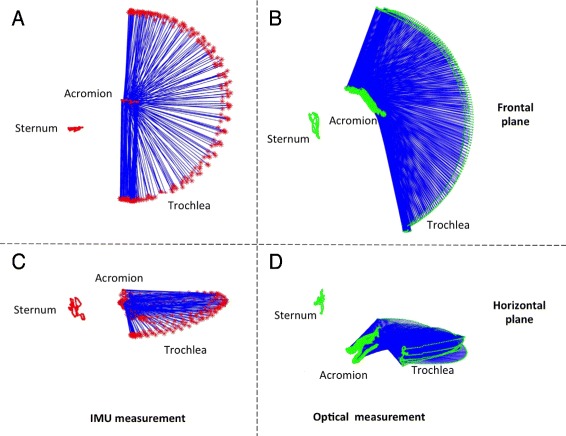
Shoulder abduction movements reconstructed by the IMU data feeding the socket-ball model (*left part*) and by the optical system (*right part*). In the *upper part*, the scapular girdle kinematic is projected on a frontal plane, in the *lower part* the projection is on a horizontal plane

**Fig. 5:**
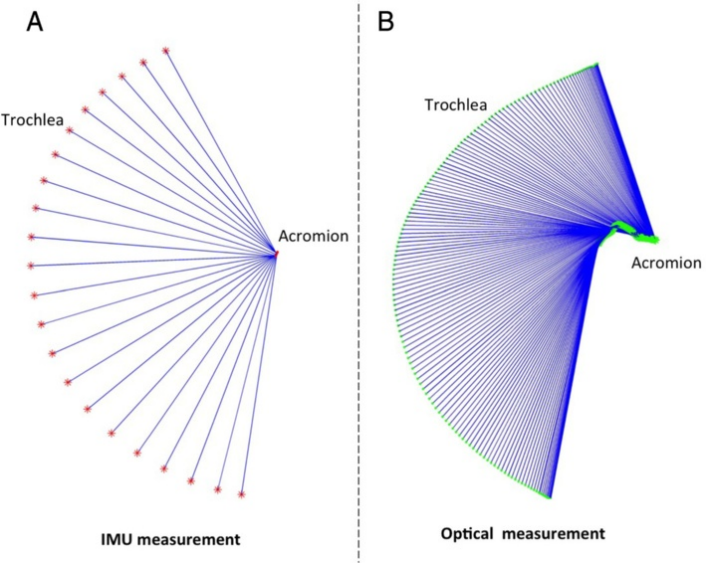
Abduction movement after the removal of the sternum oscillation. Socket-ball model fed by IMU data (*left*) and optical reconstruction (*right*)

**Fig. 6:**
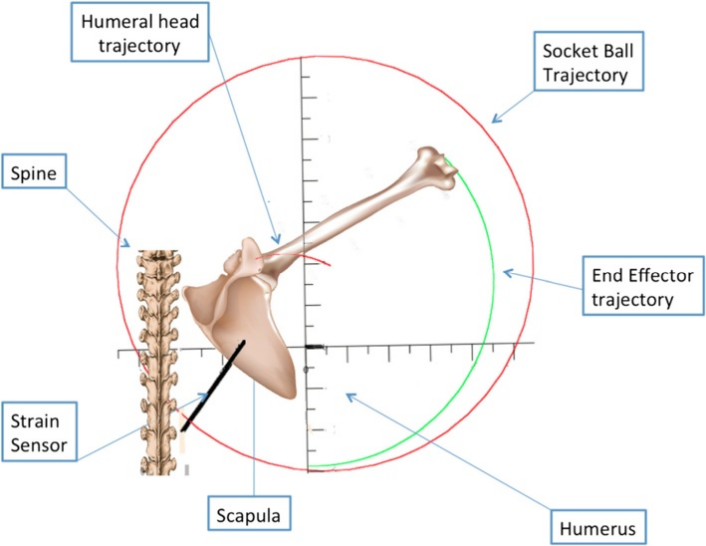
A representation of the shoulder abduction taking into account the scapular movement. The *red path* represents the acromion movement, while the *green path* is the trochlea position (End Effector). The *red circle* represents the socket-ball trajectory

The same procedure was performed to gather data on the frontal plane during an abduction. Figure 4a and c shows the trochlea trajectory obtained by the socket-ball model in the arm abduction experiments, again projected in the frontal and horizontal planes. The reference trajectories for the same movements are plotted in Fig. 4b and d. After having subtracted the sternum oscillations, Fig. 5a and b highlight the difference between the reference data and the socket-ball model (i.e. perfect circular trajectory of the trochlea). Also in this case we represented the trochlea trajectory as a generalized cycloid whose center moves on a curve which can be approximated by an elliptic arc by committing errors in the range of 1 cm. Table 2 reports the errors committed by modelling the shoulder as a socket-ball joint computed for fixed angles on the repetitions of the abduction movements. Following these considerations, we can improve the description of the double movement of the scapula and humerus according to the scapular rhythm. Figure 6 shows a frame of the estimated output, where the red line represents the trajectory of the acromion and the green line is the trochlea reference path that clearly differs from the one predicted by the socket-ball model (large circle, in red). The shoulder joint movements during the arm flexion, previously described, can be considered in analogous way. These considerations led us to the development of a 3D model which fits the collected data.

**Table 2:** Average errors and standard deviations of the socket-ball estimation as a function of the abduction angle (tests on five subjects, five times)

Abbuction angle	Error [cm]
0°	1.0±0.3
20°	3.1±0.6
40°	4.2±0.9
60°	7.8±1.2
80°	7.9±2.0
100°	9.8±1.3
120°	10.2±0.6

### Model creation and data fusion

**Fig. 7:**
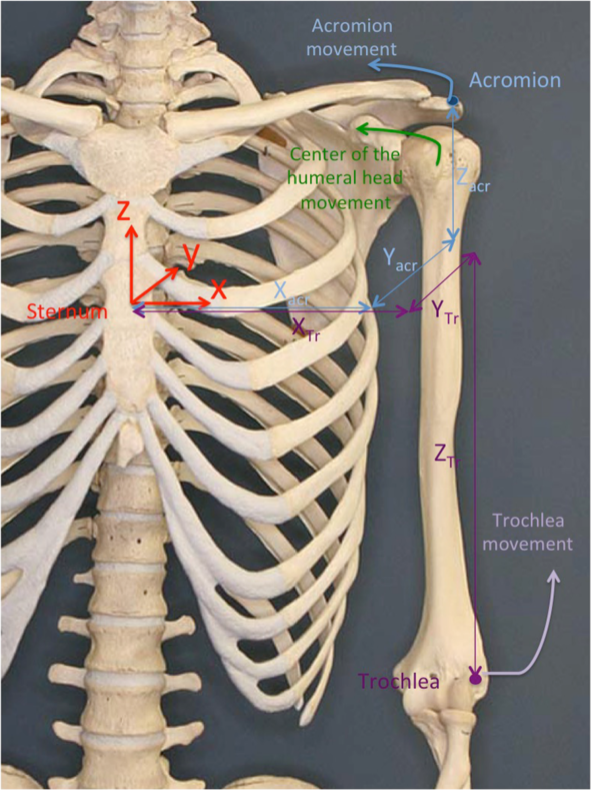
The bones of the scapular girdle, the reference frames fixed with the sternum, and the coordinates of repere points in anatomical position

Leveraging on the findings obtained for the two planar movements, we built the function that estimates the three-dimensional trajectory of the trochlea by fusing the IMUs and strain sensor outputs, formally expressed as:

(4) $$ F = \left(\begin{array}{c} \theta_{arm} \\ \psi_{arm} \\ R \end{array} \right) \mapsto \left(\begin{array}{c} X_{Tr} \\ Y_{Tr} \\ Z_{Tr} \end{array} \right)  $$

where *θ*_*arm*_,*ψ*_*arm*_ represent the horizontal flexion and abduction angles of the arm detected through the arm and sternum IMUs, *R* indicates the output of the strain sensor and *X*_*Tr*_,*Y*_*Tr*_, *Z*_*Tr*_ are the coordinates of the trochlea evaluated with respect to the sternum (see Fig. 7).

Figures 3b and 5b suggest that the acromion moves on elliptic trajectories. Thus, the 3D acromion state manifold can be described, with respect to the frame fixed with the sternum, by equation:

(5) $$ \left (\begin{array}{c} X_{acr} \\ Y_{acr} \\ Z_{acr} \end{array}\right) = \left (\begin{array}{c} A_{1,0}+A_{1,1} \sin (\theta_{a}) \cos(\psi_{a}) \\ A_{2,0}+A_{2,1} \sin (\theta_{a}) \sin(\psi_{a}) \\ A_{3,0}+A_{3,1} \cos (\theta_{a}) \end{array} \right)   $$

where *A*_1,0_, *A*_2,0_, *A*_3,0_ are the coordinates of the centre of the ellipsoid containing the acromion trajectories and *A*_1,1_, *A*_2,1_, *A*_3,1_ are the ellipsoid semi-axes. *θ*_*a*_ and *ψ*_*a*_ are the two variables that describe the scapula orientation with respect to the sternum and are dependent on *θ*_*arm*_ and *ψ*_*arm*_ through the scapular-humeral rhythm mechanism.

Assuming that the rotation center of the humeral head is in a fixed position with respect to the acromion and considering that the mutual position of the trochlea and the rotation center of the humerus remain unchanged, the trochlea coordinates can be expressed by:

(6) $$ \left(\begin{array}{c} X_{Tr} \\ Y_{Tr} \\ Z_{Tr} \end{array} \right) = \left (\begin{array}{c} B_{1}+X_{acr}+D_{ht} \sin (\theta_{arm}) \cos(\psi_{arm}) \\ B_{2}+Y_{acr}+D_{ht} \sin (\theta_{arm}) \sin(\psi_{arm}) \\ B_{3}+Z_{acr}+D_{ht} \cos (\theta_{arm}) \end{array} \right)   $$

where *B*_1_,*B*_2_,*B*_3_ represent the coordinates of the rotation centre of the humeral head with respect to a reference frame fixed with the acromion and *D*_*ht*_ represents the distance between the center of the humeral head and the trochlea. Assuming that *B*_1_,*B*_2_,*B*_3_ are negligible with respect to *A*_1,0_,*A*_2,0_,*A*_3,0_ (which are comparable with the scapula dimension), by combining Eqs. 5 and 6 we obtain:

(7) $$ {\begin{aligned} \left (\begin{array}{c} X_{Tr} \\ Y_{Tr} \\ Z_{Tr} \end{array} \right) = \left (\begin{array}{c} A_{1,0}+A_{1,1} \sin (\theta_{a}) \cos(\psi_{a})+D_{ht} \sin (\theta_{arm}) \cos(\psi_{arm}) \\ A_{2,0}+A_{2,1} \sin (\theta_{a}) \sin(\psi_{a})+D_{ht} \sin (\theta_{arm}) \sin(\psi_{arm}) \\ A_{3,0}+A_{3,1} \cos (\theta_{a})+D_{ht} \cos (\theta_{arm}) \end{array} \right). \end{aligned}}  $$

Note that, at this stage, *A*_*i,j*_ with *i*=1,2,3;*j*=0,1 and *D*_*ht*_ are unknown parameters for the model that varies among the different subjects.

To take into account the subject-dependent relation between scapular (*θ*_*a*_, *ψ*_*a*_) and gleno-humeral (*θ*_*arm*_, *ψ*_*arm*_) movements, the mechanical model proposed by Eq. 7 can be combined with the humeral-scapular rhythm relations (Eqs. 1 and 2). In addition, it is possible to introduce the information *R* derived from the strain sensor placed on the scapula. Assuming that the behaviour of the sensor is linear, its value can be related to the acromion position with respect to the frame fixed with the sternum. Under this hypothesis, we can introduce an additional constraint to the *F* determination, expressed by equations:

(8) $$ \left \{ \begin{array}{l} R= c_{0,1}+c_{1,1}\, X_{acr} \\ R= c_{0,2}+c_{1,2}\, Y_{acr} \\ R= c_{0,3}+c_{1,3}\, Z_{acr} \end{array} \right.   $$

where *c*_0,1_, *c*_1,1_, *c*_0,2_*c*_1,2_,*c*_0,3_ and *c*_1,3_ are six coefficients depending on the structure of the body of the person wearing the sensing garment. Equation 8 introduces new parameters and increases the computational cost of the identification of *F*, however it reinforces the relation between the shoulder movement and the scapular sliding. With this last step, the whole sensing system becomes redundant. This redundancy produces an overdetermined problem. Hence the solution can be found in the least-square sense by solving the variable surplus that determines the system parameters.

**Fig. 8:**
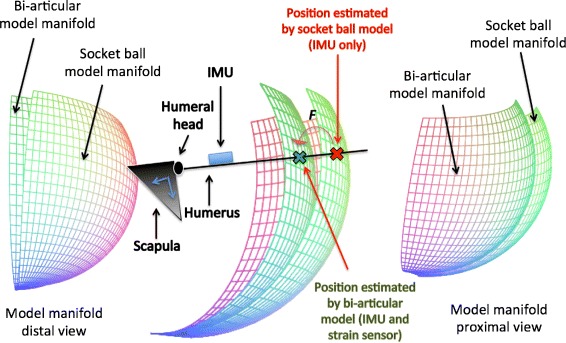
The action of *F*: the socket-ball estimation of the hand position is mapped onto the bi-articular model manifold

In summary, the construction of *F* can be obtained by considering Eq. 7 under the constraints given by Relations 1, 2 and 8. The unknown parameters *A*_*i,j*_, *D*_*ht*_ and *c*_*i,j*_ can be identified by the minimization of the following functional:

(9) $$ \begin{aligned} \mathit{G}&= \int_{I} \left(\left(X_{Tr}- \hat{X}_{Tr}\right)^{2} + \left(Y_{Tr}- \hat{Y}_{Tr}\right)^{2}\right.\\ &\qquad\qquad\qquad\qquad\quad+\left. \left(Z_{Tr}- \hat{Z}_{Tr}\right)^{2} \right) \: dI \end{aligned}  $$

on the integration domain:

(10) $$ \begin{aligned} I&=\left \{ \theta_{arm}=0,\, \psi_{arm} \in [0, 120^{\circ}] \right \} \\&\quad\cup \left \{ \theta_{arm}=90^{\circ},\, \psi_{arm}\in [0, 150^{\circ}] \right \} \end{aligned}  $$

where $\hat {X}_{Tr}$, $\hat {Y}_{Tr}$ and $\hat {Z}_{Tr}$ are the real coordinates of the trochlea derived by the optical system. The minimization of the functional 9 is executed through Lagrange’s multiplier method under the constraints, 1, 2 and 8. Symbolic computation was executed by *MapleV* to obtain formal derivatives, while numerical computation for solving Lagrange’s equations was performed using in *MATLAB*. After identifying *A*_*i,j*_*s*, *c*_*i,j*_*s* and *D*_*ht*_, the function *F* is completely determined as an iterative process which corrects the initial socket-ball evaluation through the strain sensor and the double joint movement. The status manifold of the coordinates of the trochlea is shaped on the particular subject wearing the shirt, and the creation of *F* through its parameter identification needs to be executed for each different subject using the described measurement set-up. Figure 8 shows the action of *F*. Starting from the sphere (which corresponds to the socket-ball status manifold), the bi-articular ellipsoid model described by Eq. 7 is obtained through *F*.

## Results

We assessed the performance of *F* on the five subjects in a series of upper limb vertical movements lying on different planes. The subjects repeated the movements three times.

We compared the estimated trochlea position - obtained by fusing IMUs and strain data through *F* - with the reference trajectory obtained by the optical system (trochlea marker). Table 3 reports the results of this comparison grouped in mean and standard deviation of the distance between the estimated and reference position of the trochlea, computed over the entire set of trials for each arm position. The maximal value of the average error is 1.8 cm and is obtained for 40° horizontal flexion and 120° abduction.

**Table 3:** Mean errors and standard deviations ([cm]) between the trochlea position computed via *F* and by the reconstruction done by the optical system (five individuals, three trials). Test points are spanned by *θ*=*θ*_*arm*_ and *ψ*=*ψ*_*arm*_

*θ*∖*ψ*	10°	20°	30°	40°	50°	60°	70°	80°
20°	0.4±0.6	0.4±0.3	0.7±0.4	0.8±0.3	0.5±0.2	0.7±0.3	0.5±0.4	0.5±0.2
40°	0.9±0.4	.9±0.5	0.8±0.3	1.1±0.3	0.8±0.3	0.6±0.4	0.7±0.4	0.6±0.5
60°	0.8±0.5	0.8±0.7	1.3±0.6	1.2±0.5	1.1±0.4	0.8±0.3	0.7±0.4	0.9±0.6
80°	0.7±0.2	1.1±1.1	1.2±0.5	1.5±0.7	1.3±0.5	1.1±0.7	1.0±0.7	1.1±0.7
100°	1.1±1.1	1.4±0.9	1.4±0.5	1.6±0.8	1.7±0.9	1.2±0.4	1.1±0.8	1.0±0.7
120°	1.4±0.4	1.5±1.1	1.6±1.0	1.8±0.8	1.6±1.0	1.5±1.0	1.2±0.7	1.2±0.8

In addition, to highlight the improvement of our bi-articular model, we evaluated the error of the socket-ball approach within the same set of trials. Table 4 reports the socket-ball error expressed as the mean and standard deviation of the distance between the socket-ball estimation and the reference position of the trochlea, over the entire set of trials for each arm position. As shown in Table 4, the socket-ball maximum error is greater than 11.5 cm which is 6 times higher than the bi-articular model error reported in Table 3.

**Table 4:** Mean errors and standard deviations ([cm]) between the trochlea position computed via the socket-ball model and by the reconstruction done by the optical system (five individuals, three trials). Test points are spanned by *θ*=*θ*_*arm*_ and *ψ*=*ψ*_*arm*_

*θ*∖*ψ*	10°	20°	30°	40°	50°	60°	70°	80°
20°	0.4±0.5	0.6±0.5	0.7±0.4	1.2±0.5	0.9±0.5	0.7±0.5	0.6±0.7	0.4±0.6
40°	0.8±0.6	1.1±0.7	1.2±0.5	1.2±0.5	1.2±0.4	1.1±0.6	0.8±0.6	0.6±0.4
60°	1.3±0.5	1.4±0.7	1.5±0.5	1.6±0.5	1.5±0.5	1.4±0.5	1.2±0.6	1.0±0.4
80°	2.2±1.0	2.5±0.5	2.9±0.5	3.0±0.8	2.8±0.7	2.7±0.8	2.3±0.4	2.1±0.6
100°	4.3±0.9	4.8±0.6	4.9±0.8	5.4±0.5	10.7±1.1	5.2±0.7	4.8±0.4	3.7±0.8
120°	10.3±0.7	10.3±1.0	10.6±1.2	11.5±1.1	11.3±0.9	11.0±0.6	9.8±0.9	9.2±0.7

**Fig. 9:**
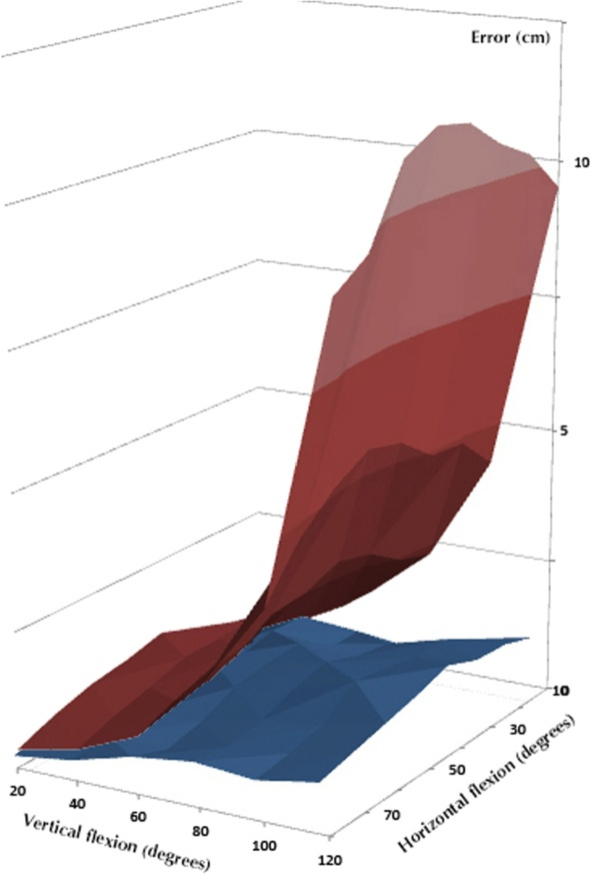
Comparison of the average errors generated by the two approaches with respect to the gold standard. The *blue surface* represents the error introduced by the bi-articular model, while the *red surface* is connected to the socket-ball and estimation of the IMUs

Figure 9 shows a visual comparison between the bi-articular and socket-ball errors, again highlighting the better performance of the estimation through F.

## Discussion

Our bi-articular model remarkably improved the reconstruction of the position of the end-effector (trochlea) with respect to the simple socket ball approximation. The measurement of the elbow kinematics, out of the scope of this research, would allow to extend these results to the estimation of the hand position in reaching activities. Considering the elbow completely extended and an average arm-length of about 80 cm, the error in hand position can be roughly extrapolated: 3.5 cm for the bi-articular model vs. 20 cm for the socket ball approximation. The bi-dimensional error map (Fig. 9) is roughly symmetrical with respect to 40° of horizontal flexion where the error is maximum (corresponding to the 40°-column of Table 3). This is due to the estimation of the model parameters described in the *Methods* section. Indeed, the integral to be minimized is defined on the domain *I* (Eq. 10) and the points of the shoulder workspace corresponding to the 40°-column are the farthest from the ones used for the identification process.

The obtained results can be considered satisfactory for a reliable monitoring of reaching activity in daily-life context. For a comparison with the recent literature, we considered the contribute of Van Meulen et al. reported in [37]. Here, the authors validated the upper limb reconstruction accuracy of the IMU-based MVN Biomech motion capturing system [33] against an optical reference system. They obtained a maximum error around 6 cm and they were able, through the described set-up, to correlate the patient’s reaching performance with the upper extremity part of the Fugl-Meyer scale [38], in a simulated in-home task. Note that MVN Biomech estimates the shoulder movement through a bi-articular approach with three IMUs, following a configuration similar to the one of Cutti et al. [13], already discussed in the *Background*.

Our methodology has the potential to be applied in monitoring the quality of the movement and evaluating the recovery obtained by adequate physical training. In stroke patients, nerve deafferentation influences the muscle control in different ways. Proximal muscles, with a cranial innervation are easier to control than distal muscles. This phenomenon can activate pathological synergies when performing activities which need to be prevented and corrected. A classical case is the replacement of the use of the deltoid muscle in anti-gravitational actions by a torque exerted by trapezius and serratus anterioris muscles. This pathological motor scheme produces an excessive rotation of the scapula to compensate for the scarce elevation of the humerus, thus violating the rhythm. Other pathological conditions that affects the scapular-humeral rhythm are the subacromial and internal shoulder impingement [39] that may represent a future objective of our analysis.

Several limitations of our work should be acknowledged. First, the upper limb reconstruction through our bi-articular model and set-up can be affected by measurement errors introduced by the textile-based strain sensor, which may present a non-negligible hysteresis [27]. A more intensive testing phase on an higher number of subjects would be needed to better quantify these aspects. However, our method is general and highlights the importance of measuring the scapular sliding independently on the particular strain sensor used (i.e. the more accurate the strain, the better the reconstruction). Secondly, the scapular-humeral rhythm - embodied in our bi-articular model - depends on the external load and this may affect our reconstruction performance. Indeed, Yoshiaki et al. demonstrated that a 3-kg load considerably modified the scapulo-thoracic vs. gleno-humeral ratio [40]. However, we are confident that for reduced loads - typical of our target application - our approximation may still be valid. Finally, the calibration procedure for the identification of the model parameters requires the acquisition of the end-effector position in planar movements through an external referenced system. This aspect may limit the potential use of our system, especially in daily life contexts where there is the need of simple and inexpensive motion tracking systems. In future works, a simplified calibration based on a reduced number of predefined positions will be investigated.

## Conclusions

This paper presented a new method for the reconstruction of the upper limb kinematics starting from minimal set of inertial and textile sensors aimed at ambulatory monitoring of the patient’s reaching activity in daily life. The idea consisted in using a bi-articular approach, describing both the gleno-humeral and scapular-thoracic joints, and in implementing a constraint given by the subject dependent ratio between the two joint’s movements, governed by the scapular-humeral rhythm. We demonstrated a reliable estimation of the hand-to-sternum position with a minimal sensing system, much less obtrusive than the existing ambulatory and multi-articular approaches.

## Ethical statement

The study protocol is a subset of a larger protocol developed within the INTERACTION (FP7-ICT-2011-7-287351) project and was carried out in accordance with the recommendations of Swiss medic, medical and ethical committee. All participants were healthy subjects and gave written informed consent in accordance with the declaration of Helsinki.
